# Study on Osseointegration Capability of β-Type Ti–Nb–Zr–Ta–Si Alloy for Orthopedic Implants

**DOI:** 10.3390/ma17020472

**Published:** 2024-01-19

**Authors:** Yu Sun, Qingping Liu, Zhenglei Yu, Luquan Ren, Xin Zhao, Jincheng Wang

**Affiliations:** 1 Orthopaedic Medical Center, The Second Hospital of Jilin University, Changchun 130041, China; yusun@jlu.edu.cn; 2 Key of Bionic Engineering, Ministry of Education, Jilin University, Changchun 130022, China; liuqp@jlu.edu.cn (Q.L.); zlyu@jlu.edu.cn (Z.Y.); lqren@jlu.edu.cn (L.R.)

**Keywords:** β titanium alloy, low elastic modulus, osseointegration, bone–implant contact

## Abstract

Osseointegration is the basic condition for orthopedic implants to maintain long-term stability. In order to achieve osseointegration, a low elastic modulus is the most important performance indicator. It is difficult for traditional titanium alloys to meet this requirement. A novel β-titanium alloy (Ti–35Nb–7Zr–5Ta)_98_Si_2_ was designed, which had excellent strength (a yield strength of 1296 MPa and a breaking strength 3263 MPa), an extremely low elastic modulus (37 GPa), and did not contain toxic elements. In previous in vitro studies, we confirmed the good biocompatibility of this alloy and similar bioactivity to Ti-6Al-4V, but no in vivo study was performed. In this study, Ti-6Al-4V and (Ti–35Nb–7Zr–5Ta)_98_Si_2_ were implanted into rabbit femurs. Imaging evaluation and histological morphology were performed, and the bonding strength and bone contact ratio of the two alloys were measured and compared. The results showed that both alloys remained in their original positions 3 months after implantation, and neither imaging nor histological observations found inflammatory reactions in the surrounding bone. The bone–implant contact ratio and bonding strength of (Ti–35Nb–7Zr–5Ta)_98_Si_2_ were significantly higher than those of Ti-6Al-4V. The results confirmed that (Ti–35Nb–7Zr–5Ta)_98_Si_2_ has a better osseointegration ability than Ti-6Al-4V and is a promising material for orthopedic implants.

## 1. Introduction

Bone replacement implants are products that replace part of bone tissue to fulfill its function. Therefore, bone implants must be mechanically stable (e.g., high strength, fatigue resistant, abrasion and corrosion resistant, etc.) and biologically stable (low cytotoxicity and good osseointegration). The definition of osseointegration was proposed by Branemark [1]. It refers to “the process resulting in direct structural and functional connection between ordered, living bone and the surface of a (load-bearing) implant” [2]. The basis of osseointegration is that the elastic modulus of the material is close to that of the bone tissue, so as to minimize the reduction in the strength of the surrounding bone tissue caused by the stress-shielding effect.

In order to find the ideal bone replacement materials, researchers have developed a series of titanium and titanium alloy materials. Among them, the most successful commercialization is the Ti-6Al-4V alloy [3,4,5,6,7,8,9,10]. Due to its good biocompatibility and relatively low elastic modulus (110 GPa), Ti-6Al-4V has become a versatile artificial joint prosthesis material. The application of Ti-6Al-4V has greatly contributed to the development of medical metal materials, but two toxic elements, vanadium (V) and aluminum (Al) are used in this type of alloy. In the 1980s, it was reported that the bone tissue around the Ti-6Al-4V artificial hip prosthesis appeared to be melanistic and infected. Studies have confirmed that V is highly toxic and can induce cancer by accumulating in various organs as a permanent implant [11,12]. After Al is absorbed, it can cause bone malacia, nervous system disorders, anemia, etc., and it may also be related to Alzheimer’s disease [7,9,13,14]. Moreover, the higher elastic modulus of Ti-6Al-4V compared to that of bone tissue (30 GPa) still cannot completely eliminate the stress-shielding effect.

Third-generation titanium alloys (β type alloys) contain biocompatible elements, such as Ti, Zr, Nb, Ta, Si, and Sn, which can also stabilize the β structure in titanium [14,15,16,17,18,19]. Overall, β-titanium alloys exhibit an elastic modulus closer to that of human bone, as well as improved biocompatibility [20,21]. In addition, the enhanced strength, plasticity, and good wear resistance properties make these alloys more suitable for use in biomedical implantation [22,23,24,25,26].

Numerous studies show that the grain size of the material has an effect on its mechanical properties. Compared with the coarse-grained material of the same composition, the fine-grained material has improved strength, plasticity, hardness, toughness, and wear resistance. La et al. [27] studied the tribological properties of Ti with different grain sizes in various environments, and the result was that ultrafine-grained Ti had better tribological properties than coarse-grained Ti. As far as biomedical alloys are concerned, in addition to better mechanical properties, fine-grained materials have better biological activity. Khang [28] and Webste [29] compared the effect of grain size on cell biocompatibility and adhesion behavior in pure titanium or titanium alloys with the same composition. The results show that the ultrafine-grained alloys and nano-alloys have higher surface energies and exhibit stronger osteoblast adhesion than the fine-grained alloys.

According to the design theory of d-electron alloy design, Li et al. have produced a new type of ultra-fine-grained titanium alloy fabricated via the spark plasma sintering (SPS) method [30]. The nominal composition is (Ti–35Nb–7Zr–5Ta)_98_Si_2_. The Ti–35Nb–7Zr–5Ta alloy system, which has been proven to have a low elastic modulus and good biocompatibility [24,31], was selected, and a Si element with improved biocompatibility was added to promote grain refinement and amorphous formation [22,31,32,33,34]. The alloy system has a low elastic modulus (as low as 37 GPa). The two-phase ultrafine microstructure includes body-centered cubic β-Ti matrix and (Ti, Zr)_2_Si reinforcement phase. Its special deformation mechanism endows it with higher strength (a yield strength of 1296 MPa and a breaking strength of 3263 MPa). Its excellent mechanical compatibility far exceeds that of the widely used Ti-6Al-4V alloy, and it has enormous application potential.

In previous studies, we verified the improved biocompatibility of this novel (Ti–35Nb–7Zr–5Ta)_98_Si_2_ alloy through in vitro studies and found that its biological activity is comparable to that of commercialized Ti-6Al-4V [35]. In this study, we further evaluated the osseointegration capacity of this alloy by implanting the material into the bones of rabbits.

## 2. Experimental

### 2.1. Preparation of TiNbZrTaSi Specimens

**Figure 1:**
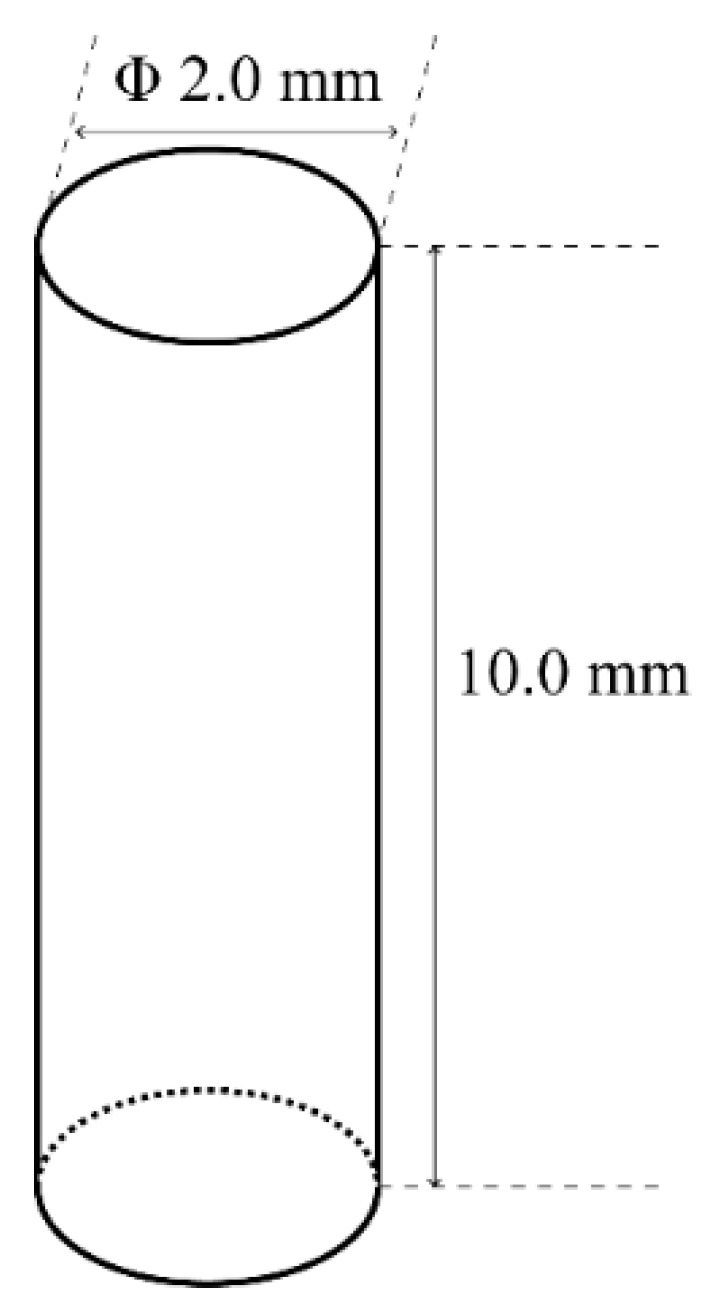
Geometry of the implants.

(Ti–35Nb–7Zr–5Ta)_98_Si_2_ alloy was prepared as previously described [30]. Briefly, high-purity powders of Ti, Nb, Zr, Ta, and Si were mixed in a mechanical mixer for 4 h at 100 rpm. Subsequently, the mixture was subjected to high-energy ball milling at a rotational speed of 4.1 s^−1^ under the protection of purified argon gas. After grinding, the powder sample was loaded into a graphite mold and sintered on an SPS-825 system (Sumitomo Coal Mining Co., Ltd., Tokyo, Japan). The alloy was sintered at a vacuum of 1233 K at a pressure of 50 MPa for 5 min. The sintered bulks were cut into cylindrical specimens (Φ2 mm × 10 mm), as shown in Figure 1. The commercial titanium alloy Ti-6Al-4V (AK Medical Co., Ltd., Beijing, China) was used as the control group. All specimens were polished, ultrasonically, cleaned with distilled water, an acetone solution, and 70% ethanol for 20 min. Then, they were ultrasonicated with distilled water for 15 min, autoclaved at 121 °C for 40 min, and vacuum-dried. SEM images showed that the polished (Ti–35Nb–7Zr–5Ta)_98_Si_2_ and Ti-6Al-4V alloy samples had glossy surfaces and scratches in the same direction [35].

### 2.2. Surgical Procedures

The animal experiment was approved by the Ethical Committee for Animal Experiments of Jilin University of China. Six New Zealand white rabbits were used in the experiments, with no regard to gender. (Ti–35Nb–7Zr–5Ta)_98_Si_2_ and Ti-6Al-4V were implanted into the left and right femurs of rabbits, respectively, and three specimens were implanted into each femur. The specific surgical methods were as follows: animals were placed in prone position, and 3% sodium pentobarbital was injected into the ear vein at a rate of 1 mL/kg for anesthesia. The left and right hind limbs were shaved to expose the skin at the femur, sterilized with iodine, and a sterile towel was laid out. The skin and subcutaneous tissue were incised, muscles were separated, and the periosteum was incised to expose the femoral shaft. On the lateral side of the femoral shaft, three holes with a diameter of 1.8 mm were drilled a low-speed drill in sequence, with a depth of about 10 mm and a hole spacing of 10 mm. During drilling, the local tissue was cooled with normal saline to prevent high temperature necrosis of the surrounding bone tissue. The specimens were inserted in the hole. After the surgery, the wound was subcutaneously closed with 4-0 nylon sutures. In the animal facility of the Laboratory Animal Center of Jilin University in China, the rabbits were housed in separate metal cages and moved freely in their cages. These animals were given specialized food and had access to water at will. Basic biological functioning, eating and excretion, behavioral signs associated with postoperative pain were carefully checked daily, and suture care for postoperative infection and surgical wounds, bleeding, and/or infection were monitored. After surgery and during the following three days, the animals received penicillin (8000 iu, qd, i.v.) and a 2% solution of tramadol hydrochloride (1.0 mg/kg, q12h, s.q.). The skin sutures were removed from the wound within ten days after the implantation surgery.

### 2.3. Imaging Observation

X-ray films were taken three months after the specimens were implanted into the animals’ femurs, to evaluate the location of the implanted specimens in the bone and their surrounding tissue.

### 2.4. Osseointegration Strength

After the X-ray films were taken, the animals were sacrificed by overdose of 3% pentobarbital. The femurs were exposed, and a visual observation was conducted to determine the presence of redness, purulence, necrosis, and a gap between the implants and the surrounding tissue. The femurs were removed and the femur specimens of three animals were used for hard tissue sectioning and staining, while the other specimens were used for push-out experiments.

**Figure 2:**
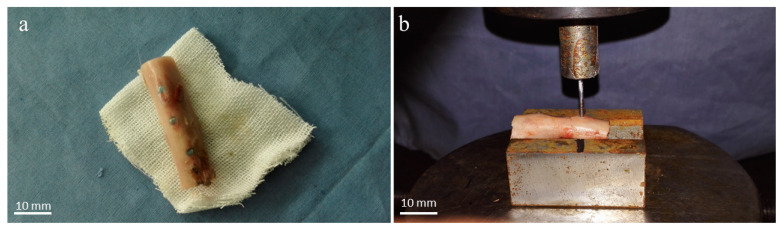
Push-out test operation. (**a**) The femur sample containing implants. (**b**) The implanted specimens were pushed axially with a 1.8 mm diameter thimble.

Both ends of the femurs were cut off with a diamond wheel (Figure 2a), and the femoral samples were put on a metal base with a V-groove for fixation. The implanted specimens were pushed axially with a 1.8 mm diameter thimble (Figure 2b). The mechanical push-out experiment was carried out on a universal mechanical machine. The loading speed was set to 1 mm/min, and the force-offset curve was recorded.

### 2.5. Histological Preparation

Specimens were processed using methacrylate embedding techniques [36].

The rabbit femurs with alloy specimens were fixed in 10% formalin for two weeks and then dehydrated with alcohols at different concentrations (70%, 80%, 96%, 99.8%, respectively) under constant agitation.

Plastic permeation was carried out under continuous agitation (Exakt 510) and under vacuum according to the following procedure: (1) Technovit 7200^®^/BPO:alcohol = 30:70. (2) Technovit 7200^®^/BPO:alcohol = 50:50. (3) Technovit 7200^®^/BPO:alcohol = 70:30. (4) Technovit 7200^®^/BPO. (5) Technovit 7200^®^/BPO. Each procedure was carried out for three days.

The specimens were light-cured with Technovit 7200 VLC solution (Morphisto, Offenbach am Main, Germany) in the EXAKT E520 light-curing embedding machine (EXAKT Advanced Technologies GmbH, Norderstedt, Germany) for 12 h, cut into sections with a thickness of about 200 μm with the EXAKT E300CP hard tissue microtome, and then polished with 800 mesh, 1200 mesh, and 4000 mesh sandpaper using the EXAKT E400CS tissue grinder to make sections with a thickness of about 70 μm for staining.

### 2.6. Histomorphology

The sections containing Ti-6Al-4V and (Ti–35Nb–7Zr–5Ta)_98_Si_2_ were stained with HE, Masson, and toluidine blue, respectively. The bone-to-implant contact (BIC) rate was measured by an image information management software, Image-Pro Plus vision 6.0 (MediaCybernetic, Silver Springs, MD, USA).

### 2.7. Statistical Analysis

Results were presented as the mean ± standard deviation. Differences between treatment groups were analyzed using a *t* test. Differences with *p* < 0.05 were considered statistically significant.

## 3. Results

### 3.1. Imaging Observation

**Figure 3:**
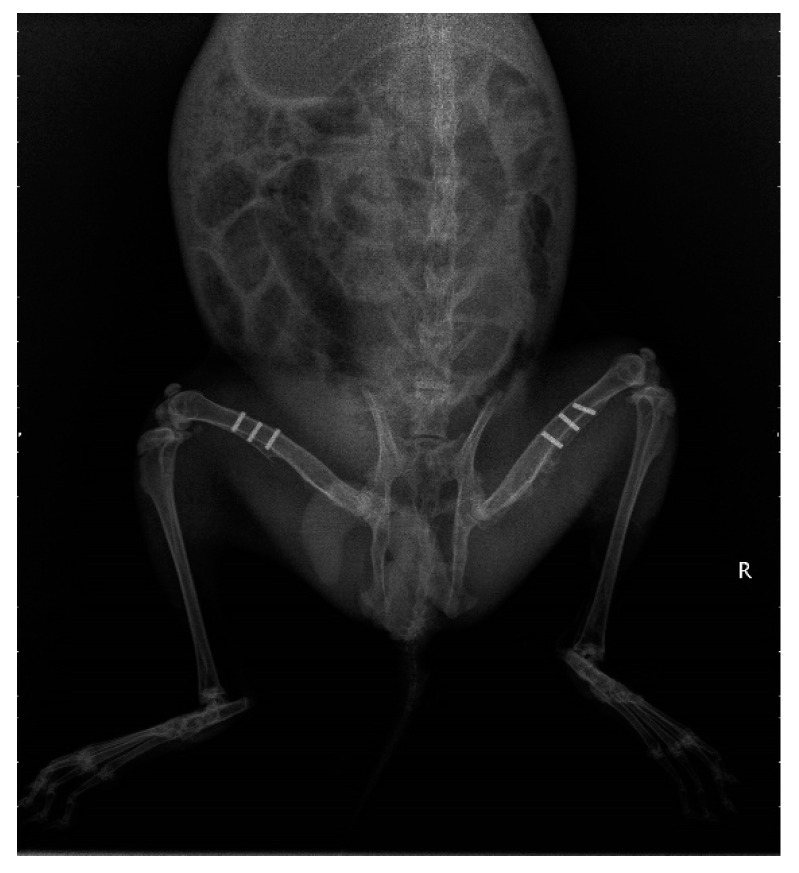
X-ray image of rabbit femurs. R: right side of the image.

All of the experimental animals were recovered from the implant surgery without any operative or postoperative complications. X-rays can show the location of the implant and the condition of the surrounding bone. It is shown in Figure 3 that all the Ti-6Al-4V and (Ti–35Nb–7Zr–5Ta)_98_Si_2_ specimens were in their original positions without displacement. No obvious inflammatory reaction, osteoporosis, and lucid zone were observed in the surrounding bone of the specimens. At the same time, a periosteal reaction could be observed around the specimens.

### 3.2. Osseointegration Strength

**Figure 4:**
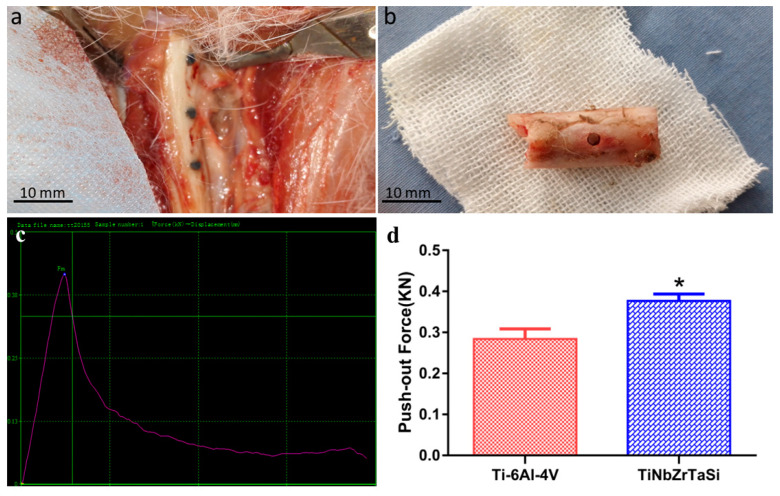
(**a**) The exposed femur containing implants three months after operation. (**b**) The femur sample after the implants were pushed out. (**c**) The force-offset curve. (**d**) The maximum pushing force analysis of (Ti–35Nb–7Zr–5Ta)_98_Si_2_ and Ti-6Al-4V. (* *p* < 0.05).

The femurs were exposed three months after the operation (as shown in Figure 4a). There was no local reaction such as redness, purulence, and tissue necrosis around the specimens. All the specimens were well integrated with the bone tissue, and their surfaces were covered with periosteum. No obvious gaps were observed.

In most studies, the maximum force during the push-out process is an indicator of the bond between the implant and the tissue. The force-offset curve showed in Figure 4c exhibited linearity before the maximum force point, and declined after the specimens were pushed out at the maximum force point. Figure 4d shows the statistical analysis results of the maximum pushing force of the two alloys. It can be observed that the maximum pushing force of (Ti–35Nb–7Zr–5Ta)_98_Si_2_ (377 N ± 17 N) was greater than that of Ti-6Al-4V (284 ± 25 N), and the difference between the two forces was significant (*p* < 0.05).

### 3.3. Histomorphology

**Figure 5:**
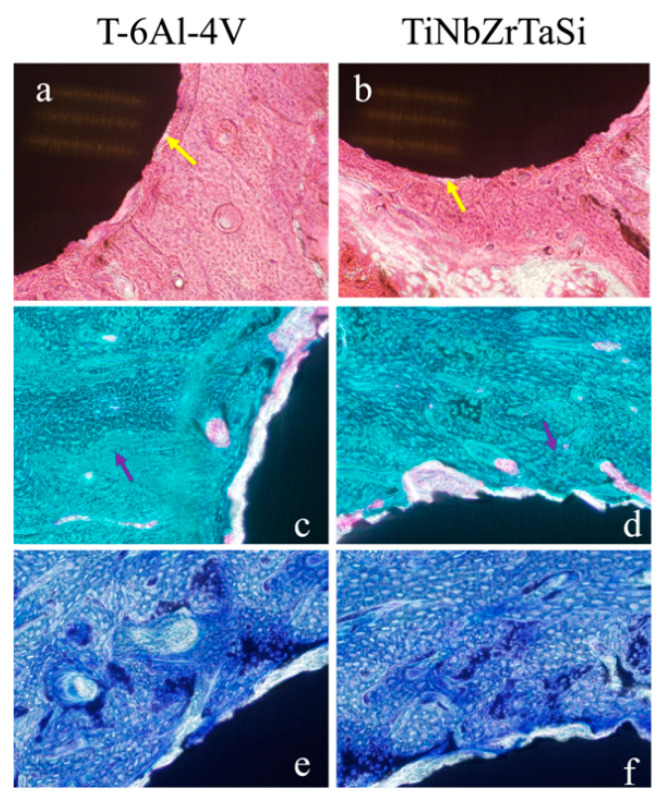
(**a**,**b**) HE staining sections of Ti-6Al-4V (**a**) and (Ti–35Nb–7Zr–5Ta)_98_Si_2_ (**b**). Yellow arrows: gaps between implants and bone tissue. (**c**,**d**) Masson staining sections of Ti-6Al-4V (**c**) and (Ti–35Nb–7Zr–5Ta)_98_Si_2_ (**d**). Purple arrows: new bone tissue. (**e**,**f**) Toluidine blue staining sections of Ti-6Al-4V (**e**) and (Ti–35Nb–7Zr–5Ta)_98_Si_2_ (**f**).

HE staining, Masson staining, and toluidine blue staining were used to evaluate the tissue response around the implanted specimens and to measure the BIC rate. It can be observed from the HE-staining images that all specimens, whether (Ti–35Nb–7Zr–5Ta)_98_Si_2_ or Ti-6Al-4V, were surrounded by extensive bone tissue. There was no obvious fibrosis and cysts. No material fragments and inflammatory cell infiltration were found at the implant–bone interface (Figure 5a,b).

Masson staining can distinguish collagen and muscle fibers well and observe the maturity of bone tissue. It can be seen from the Masson staining photos that the implanted specimens were surrounded by a large amount of new bone tissue, and no fibrous tissue infiltration was observed in the implant–bone interface (Figure 5c,d).

Toluidine blue staining verified the results of Masson staining. In the toluidine blue-staining images, the new bone tissue dominated by osteoid appeared light blue, while the mineralized bone tissue and the specimens appeared dark blue and black, respectively (Figure 5e,f).

**Figure 6:**
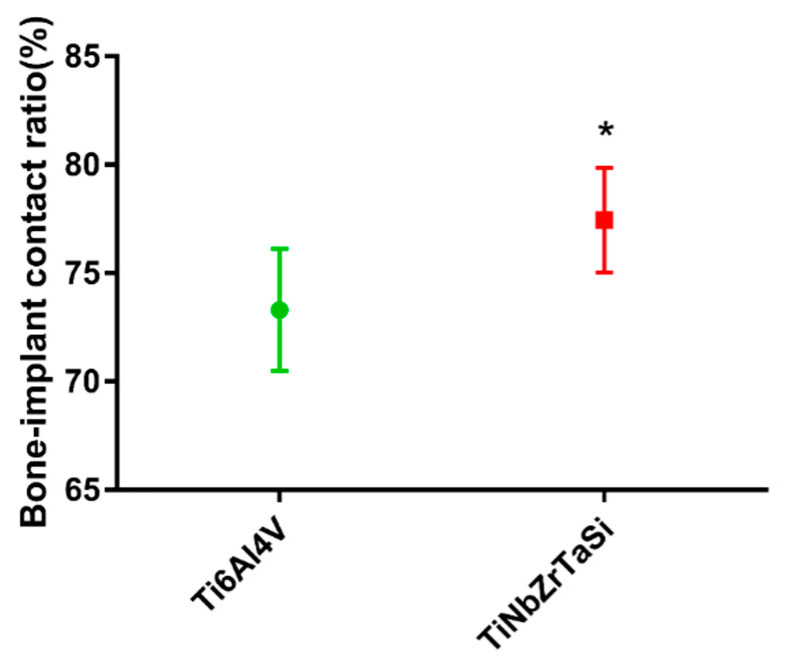
The BIC ratio of Ti-6Al-4V and (Ti–35Nb–7Zr–5Ta)_98_Si_2_ (* *p* < 0.05).

The BICs of the two samples were compared using the image analysis software Image-Pro Plus vision 6.0 (MediaCybernetic, Silver Springs, MD, USA). The 77.45% of (Ti–35Nb–7Zr–5Ta)_98_Si_2_ was higher than the 73.31% of Ti-6Al-4V (Table 1), which was confirmed via statistical analysis to be significant (Figure 6) (* *p* < 0.05).

## 4. Discussion

In the process of manufacturing long-term functional implants, biocompatibility and long-term stability are the primary considerations, and the latter needs to be achieved through good osseointegration. In a previous study, we verified the good biocompatibility of the newly designed titanium alloy (Ti–35Nb–7Zr–5Ta)_98_Si_2_ through in vitro tests [35]. This study further evaluated the osseointegration properties of this alloy.

Osseointegration refers to the persistent bony contact between viable bone tissue and an implant. Branemark reported that the implanted pure titanium metal was unusually strongly bound to rabbit bone when studying the microcirculation in the bone marrow cavity and gave this definition for the first time to distinguish it from fibrous integration in histological characteristics [1]. The study of osseointegration involves a variety of methods, each with its own merits [37], and the comprehensive application of different methods can provide a more complete and detailed understanding of the tissue response and structural changes of the material–bone interface. Among them, X-ray, optical microscopy, histological analysis, biomechanical testing, etc. are more commonly used. In this study, X-ray, biomechanical, and histological analyses were selected to study the osseointegration of the interface between the materials and the bone tissue.

As a non-invasive test, X-rays can be used to observe the bone-material interface without causing damage to the animals. It can distinguish between bone, fibrous tissue, and inflammation in surrounding tissues by grayscale values. In this study, no gap was observed between the implant and the bone tissue. The bone tissue was directly bound to the materials, with no light transmission area being seen. And the surrounding tissue showed no signs of decreased bone density, demonstrating that the implant formed good osseointegration with the bone tissue and did not cause inflammatory changes or fibrous osteogenesis (Figure 3).

Theoretically, we can consider biomechanics to be the gold standard for measuring the degree of osseointegration. The better the degree of osseointegration, the stronger the bond between the material and the bone. The pull-out test is generally used for cylindrical implants inserted in the proximal and distal portion of long bones [38]. In this study, the push-out test was selected as a means to detect the bonding strength of (Ti–35Nb–7Zr–5Ta)_98_Si_2_ alloy and the bone interface, and the maximum push-out force was used as the criterion for evaluating the bonding strength (Figure 4). The maximum push-out forces of (Ti–35Nb–7Zr–5Ta)_98_Si_2_ alloy and Ti-6Al-4V alloy reached 377 N and 284 N, respectively, indicating that the (Ti–35Nb–7Zr–5Ta)_98_Si_2_ alloy, whose elastic modulus is closer to that of human bone tissue, can effectively avoid the stress-shielding effect, promote the growth of surrounding bone tissue, and ensure the strength of the bone. In contrast, the biocompatibility and biological activity of the (Ti–35Nb–7Zr–5Ta)_98_Si_2_ alloy and the Ti-6Al-4V alloy were compared in our previous study, and the results confirmed that there was no significant difference between the two alloys [30]. The difference in the bonding strength of the two alloys should be attributed to differences in elastic modulus. Because the elastic modulus (37 GPa) of the TiNbZrTaSi alloy is closer to that of human bone tissue (30 GPa), its stress conduction is more even; thus, the surrounding bone tissue grows and rebuilds under stress, showing higher bonding strength [39].

Hard tissue sections can be used to make sections of bone tissue containing metal specimens without prior decalcification. The implant remains in situ, and the section can reflect the growth of the peri-implant bone tissue without destroying the original tissue structure of the implant–bone interface. We assessed the tissue response of the implant–bone interface through histomorphological observation and explored the reasons for the difference in the osseointegration strength of the two alloys. Hard tissue sections were stained with HE staining, Masson trichrome staining, and toluidine blue staining to observe the growth of inflammatory cells, osteoblasts, osteoclasts, fibrous tissue, and new bone. The BIC rate can be measured on histological sections, allowing for an intuitive evaluation of the ability of the material to directly bind to bone tissue. It can be observed from the HE-staining images that, although there was a partial gap at the implant–bone interface, no obvious inflammatory cell infiltration was observed (Figure 5a,b). This result was consistent with that from the previous study, in which we confirmed that none of the alloys caused the secretion of the inflammatory factor interleukin-6 (IL-6) [35]. Masson staining was mainly used to distinguish bone tissue and muscle fiber tissue and to evaluate the maturity of bone tissue. The results showed (Figure 5c,d) that after three months of implantation, the implants were surrounded by a large amount of new bone tissue, and no muscle fiber invasion was found in the implant–bone interface, indicating that the connection between the two was mainly bony rather than fibrous. Toluidine blue staining-images (Figure 5e,f) also showed massive new bone formation around the implants.

We measured the BIC rate on histological sections to verify the osseointegration effect of the two alloys. It can be observed (Figure 6) that the BIC rate of (Ti–35Nb–7Zr–5Ta)_98_Si_2_ (77.45%) was greater than that of Ti-6Al-4V (73.31%), and the difference was significant. According to the definition of osseointegration, more osseous connections between the implant and the surrounding bone and fewer fibrous connections lead to higher strength of the interface. This results in a more balanced biomechanical environment surrounding the implant and a better long-term stability of the implant. The higher BIC of (Ti–35Nb–7Zr–5Ta)_98_Si_2_ was consistent with the result of higher maximum push-out force. In the present study, the (Ti–35Nb–7Zr–5Ta)_98_Si_2_ alloy exhibited a larger BIC than the Ti-6Al-4V alloy, which in turn resulted in a higher osseointegration strength. This result was consistent with those of other related studies. Lin et al. [40] reported more new bone formation on the surface of low-elastic titanium alloys than high-elastic titanium alloys. Simon et al. [41] confirmed that low-elastic titanium alloys can promote good stress transfer, enhance the osseointegration of the implant, reduce bone resorption, and avoid the occurrence of prosthesis loosening. Stoppie et al. [42] used finite element analysis to confirm that the osseointegration rate and new bone thickness of the low-elastic implant were significantly higher than those of the high-elastic implant.

Bone remodeling is a long-term process, i.e., the occurrence of bone resorption and new bone formation is dynamic. The remodeling process can be clearly observed in the peri-implant area 6–12 weeks after implant installation. Most of the gaps are filled with mineralized bone, a mature bone tissue that is in close contact with the implant surface, resulting in successful osseointegration. The skeletal response in humans and animals resembles load-induced bone remodeling that follows Wolf’s Law, where bone tissue adapts to the load (or lack thereof) it is subjected to [43].

Obviously, the stresses and strains in the bone around the interface will counteract the load on the implant [2]. High interfacial stresses favor the formation of fibrous tissue and are associated with peri-implant bone damage. In addition, high interfacial stress can lead to increased micromotion at the implant–bone interface [44], resulting in nonmineralized fibrous tissue encapsulation [45], thus influencing osseointegration and bone remodeling [46,47]. Plenty of experimental studies have confirmed that a mismatch between the elastic modulus of the implant material and the bone tissue will cause the stress to be concentrated within the material itself as it passes through the implant. This stress cannot be well dispersed and transmitted to the bone tissue. This phenomenon is known as stress shielding [48,49,50]. Hedia [51] reported that a reduction in the modulus of elasticity can reduce stress concentrations. Therefore, it is reasonable to believe that the better osseointegration performance of the TiNbZrTaSi alloy in this study should be due to its relatively low elastic modulus.

Although there are important discoveries revealed by this study, there are also limitations. First, in this study, although we implanted three (Ti–35Nb–7Zr–5Ta)_98_Si_2_ alloys and three Ti-6Al-4V alloys in the two femurs of each rabbit, respectively, only nine samples were used for histological analysis and push-out test for each alloy. The sample size was relatively small compared to that of other studies; thus, false-positive results may appear. Secondly, although the surface treatment and morphology of the two metals were briefly described in this study, the surface roughness data could not be provided. This is a flaw in our study design. In summary, further improved research is necessary. First, the sample size needs to be enlarged. Second, control groups with different surface roughness need to be set up to exclude the effect of surface roughness on osseointegration results as much as possible.

Notwithstanding its limitation, this study does suggest that this new alloy with good biocompatibility [35] and a very low elastic modulus(~37 GPa vs. ~30 Gpa of bone vs. ~110 GPa of Ti-6Al-4V) [14,30] has improved osseointegration ability and is a promising orthopedic implant material.

## 5. Conclusions

This new β-type titanium alloy with both high strength and a low elastic modulus has good biocompatibility. After the specimens were implanted in vivo, no inflammatory response of the surrounding tissue was triggered. When compared with Ti-6Al-4V, (Ti–35Nb–7Zr–5Ta)_98_Si_2_ exhibited higher osseointegration strength and BIC. Since we previously demonstrated that the bioactivities of the two alloys are similar, the improved osseointegration behavior of (Ti–35Nb–7Zr–5Ta)_98_Si_2_ should be attributed to its low elastic modulus. This alloy may be a superior implant material for biomedical application.

## Figures and Tables

**Table 1:** The BIC ratio of Ti-6Al-4V and (Ti–35Nb–7Zr–5Ta)_98_Si_2_.

Implant	BIC (%)
Ti-6Al-4V	73.31 ± 1.07
(Ti–35Nb–7Zr–5Ta)_98_Si_2_	77.45 ± 0.91 *

* *p* < 0.05.

## Data Availability

Data are contained within the article.
